# Supplementary material for: Unraveling job demand-control-support patterns and job stressors as predictors: Cross-sectional latent profile and network analysis among Italian hospital workers
Source: PLoS One. 2025 Jun 9;20(6):e0325528. doi: 10.1371/journal.pone.0325528 (PMC12148104; doi:10.1371/journal.pone.0325528)
Supplement: Attachment — Submitted filename: response to reviewers.docx [file pone.0325528.s002.docx]

Dear Dr. Zhang,

Thank you for the opportunity to revise our manuscript.

We are grateful for the time and effort reviewers dedicated to reviewing our manuscript. Comments and suggestions were insightful and greatly improved the clarity and overall quality of our paper. We have carefully addressed each point, and we provide a detailed response below.

Each Reviewers’ points are reported in bold followed by our answers.

**Editor's Comments:**

1. **A bit more depth is needed in terms of the literature review with a particular emphasis needed on the most up-to-date (years 2023-2024) management theories in the area so that you can build the agreement a bit more for why your manuscript is original by filling a gap in the research and how the paper makes a significant contribution.**

(lines 70-115) - This is an interesting point that needs some clarification. At this point, no recent management theories have been developed on job stress and latent profile analysis. In this sense, we focused on the research gap on these topics. We have expanded the literature review strengthening the rationale for our study and highlights its contribution to the field by demonstrating how our person-centered approach can address gaps in understanding job stress within the context of the JDCS model.

2. **Grammar and Writing Style:** We have thoroughly reviewed the manuscript for grammar, typos, and writing style issues. We have corrected all identified errors and improved the overall clarity and flow of the text.

**Reviewer #1:**

1. **Justification of the Topic:** We have expanded the introduction to provide a more detailed justification of the topic, emphasizing the limitations of traditional variable-centered approaches and the potential benefits of using a person-centered approach to understand job stress profiles (lines 70-115).
2. **Ethics Statement:** We have added a statement in the Methods section confirming that the study was approved by the relevant bioethics committee (lines 219-221).
3. **English Expression:** We have carefully reviewed the manuscript for English language and expression issues and have made necessary corrections to improve clarity and accuracy.

**Reviewer #2:**

1. **Examples of Items:** We have provided examples of items for different variables (lines 223-228) to enhance reader understanding.
2. **Typographical Error:** We have corrected the typographical error (line 309) 0.840 to 0.940.
3. **Data Sum:** We have reviewed the data and corrected the sum to 100% on page 12, lines 280-289.
4. **Citation Consistency:** We have ensured consistency in the citation of literature throughout the manuscript, including correcting the citation on page 14, line 322.
5. **Capitalization:** We have corrected the capitalization of "Participatory Leader" to "participatory leader" on page 18, line 414.
6. **DOIs:** We have added DOIs for all cited literature where available.
7. **Citation Order and Missing Citation:** We have reviewed and corrected the order of citations in the text and have added the missing citation for literature 13.
8. **Use of "We":** We have reviewed the use of "we" throughout the manuscript and have replaced it with the neutral form where appropriate.

We believe that these revisions have significantly strengthened the manuscript and addressed all concerns raised by the reviewers.

Thank you for your time and consideration.

Sincerely,

Igor Portoghese
