# Supplementary material for: Unraveling job demand-control-support patterns and job stressors as predictors: Cross-sectional latent profile and network analysis among Italian hospital workers
Source: PLoS One. 2025 Jun 9;20(6):e0325528. doi: 10.1371/journal.pone.0325528 (PMC12148104; doi:10.1371/journal.pone.0325528)
Supplement: Attachment — Submitted filename: response_to_reviewers_auresp_2.docx [file pone.0325528.s003.docx]

Dear Dr. Zhang,

Thank you for the opportunity to revise our manuscript. We are grateful for the time and effort reviewers dedicated to reviewing our manuscript. We found reviewers’ comments and suggestions very insightful.

We have carefully addressed each point, and we provide a detailed response below.

**Reviewer #3:**

**In the Ethics Statement subsection, the Authors state: “Anonymity was assured because no IP address was registered, and no sensitive data were requested.” I assume the authors meant "personal data" rather than "sensitive data." For a study to be truly anonymous, no personal data should be collected, regardless of its sensitivity.**

- We agree with the reviewer’s comment, and we changed it as suggested.

**Furthermore, the Authors write: "We can share only the anonymized dataset due to ethics rules." Please note that anonymous data cannot be anonymized. I assume the Authors aim to protect participant anonymity by not providing sociodemographic or work-related variables, which, when combined, might compromise anonymity in some cases. If this is the case, the authors should clarify this explicitly**.

- We updated the data availability statement as follows:
  “The data analyzed in this manuscript have been posted in Open Science Framework (osf.io) and can be accessed from the following DOI: 10.17605/OSF.IO/NV6DK.”

**In the Abstract, it is unclear whether the full sample (N = 1,464) was used for the network analysis.**

- We moved the sample detail to the beginning of the sentence. All analyses were performed using the entire sample (N = 1,464).

**There is an inconsistent capitalization in the title (why are "job" and "hospital" capitalized?).**

- We fixed it. Thank you for carefully reading our manuscript.

**I noticed some typos in the manuscript—the Authors are requested to correct them.**

- We have carefully proofread the manuscript and fixed any typos.

**The utility of network analysis for this study should be discussed in more detail. While the Authors apply network analysis alongside latent profile analysis, it would be helpful to elaborate on why this approach was particularly useful for understanding job demand-control-support patterns. Specifically, the manuscript should clarify what unique insights network analysis provides beyond traditional statistical methods as well as latent profile analysis**.

- We agree with the reviewer’s point. The rationale behind the integration of LPA and network analysis was weak. We rewrote the paragraph by clarifying the unique value of integrating LPA and network analysis in job stress. In addition, as we have placed more emphasis on network analysis, we have added an additional network-related limitation in the discussion of limitations to the study. It is important for the reader to understand the exploratory nature of our study and the possibility that the results may be flawed by methodological limitations. Too much emphasis on adopting this methodology could create the false expectation that this is an analysis without limitations.

**The Authors state: “Despite the recent call for adopting person-centered approaches in occupational research [20], the application of this methodology is still in its infancy.” However, I would not characterize person-centered approaches as being in their infancy. Please refer to Spurk et al. (2020) for a review on this topic**.

- We rephrased the sentence highlighting that the use of that methodology within occupational research is still in its infancy.

**It would be beneficial to explain in more detail why role clarity and workplace relationships were chosen as predictors of latent profiles**.

- We agree with the reviewer’s comment. Thus, we expanded this section, adding the rationale behind this choice. However, it is important to highlight that our study was explorative in its nature and a very limited number of studies have considered predictors of JDCS profiles.

**When describing the number of profiles identified in previous studies, the authors write:
“For example, Charzyńska et al. [21] identified five profiles, whereas Portoghese et al. [16], Knight et al. [22], Urbanavičiūtė and Lazauskaitė-Zabielskė [23], Min and Hong [24], and Marzocchi et al. [25] identified four profiles. Finally, Santos et al. [26] and Galbraith et al. [25] identified three profiles.”
However, since different indicators (both quantitative and qualitative) were used in these studies, they are not directly comparable without reservations. This should be clearly stated in the manuscript, or the authors are encouraged to provide more details on the cited studies.**

- We agree with the reviewer’s point. We added a sentence that remarks that: “Most of these studies considered different profile indicators and different theoretical models (JDC, JDCS, and JD-R), limiting the consistency of the results.”

**The sentence “We considered profile 1 (isolated prisoner) to be the least favorable reference for profile comparison” is unclear. The Authors are asked to rephrase it for clarity**.

- To avoid confusing the reader, we have removed “be the least favorable” from the sentence

**Finally, I would appreciate a more detailed discussion of the practical implications of this study.**

- We have rewritten the practical implications of this study.

We believe that these revisions have significantly strengthened the manuscript and addressed all concerns raised by the reviewers.

Thank you for your time and consideration.

Sincerely,

Igor Portoghese
